# Distinct patterns of mutational sensitivity for *λ* resistance and maltodextrin transport in *Escherichia coli* LamB

**DOI:** 10.1099/mgen.0.000364

**Published:** 2020-04-02

**Authors:** Bryan Andrews, Stanley Fields

**Affiliations:** ^1^​ Molecular and Cellular Biology Program, University of Washington, Seattle WA, USA; ^2^​ Department of Genome Sciences, University of Washington, Seattle WA, USA; ^3^​ Department of Medicine, University of Washington, Seattle WA, USA

**Keywords:** *Escherichia coli*, LamB, phage* λ*, evolution, nutrient transport

## Abstract

Bacteria can evade cohabiting phages through mutations in phage receptors, but these mutations may come at a cost if they disrupt the receptor’s native cellular function. To investigate the relationship between these two conflicting activities, we generated sequence–function maps of *Escherichia coli* LamB with respect to sensitivity to phage *λ* and transport of maltodextrin. By comparing 413 missense mutations whose effect on both traits could be analysed, we find that these two phenotypes were correlated, implying that most mutations affect these phenotypes through a common mechanism such as loss of protein stability. However, individual mutations could be found that specifically disrupt *λ*-sensitivity without affecting maltodextrin transport. We identify and individually assay nine such mutations, whose spatial positions implicate loop L6 of LamB in *λ* binding. Although missense mutations that lead to *λ*-resistance are rare, they were approximately as likely to be maltodextrin-utilizing (Mal^+^) as not (Mal^-^), implying that *E. coli* can adapt to *λ* while conserving the receptor’s native function. We propose that in order for *E. coli* and *λ* to stably cohabitate, selection for *λ*-resistance and maltose transport must be spatially or temporally separated.

## Data Summary

All raw sequencing reads are available in the National Center for Biotechnology Information (NCBI) Sequence Read Archive (SRA) linked to BioProject PRJNA604031 (https://www.ncbi.nlm.nih.gov/bioproject/604031). Read counts, scores for all mutations and growth curves are available via figshare: (https://doi.org/10.6084/m9.figshare.c.4837605.v1).

**Impact Statement**

Bacteria rapidly adapt to changes in their environment. Studying the costs of these adaptations is critical to understand how bacteria evolve in their native communities, and potentially for developing strategies to inhibit the growth of pathogenic bacteria. We focus on the *Escherichia coli* protein LamB, which plays dual roles, one as the receptor for phage *λ* and another as the transporter of maltose-related sugars. We generate large numbers of mutations in this protein and assess their effects on both λ infectivity and maltose transport by using a DNA sequencing-based readout. We discover rare mutations that allow *E. coli* to acquire resistance to *λ* without the cost of their losing maltose transport. These mutations disrupt a common structural feature, loop L6, of the LamB protein and identify this feature as a likely determinant of *λ* binding. The frequency of these mutations implies that in the wild, simultaneous selection for both *λ*-resistance and maltose transport should uncover mutations that benefit the bacteria both ways. However, if selections for *λ*-resistance and maltose transport are spatially or temporally segregated from each other, evolution may nonetheless occur by initially producing a loss-of-function mutation followed by back mutation or compensatory mutation to a protein that transports maltose.

## Introduction

Individual proteins frequently carry out multiple distinct activities. When pathogenesis is involved, two activities can be in conflict, with one benefitting the host cell and the other the pathogen. For instance, in the case of some host-cell surface proteins, one activity may be to transport a nutrient while another is to serve as the receptor for a virus [1, 2]. The cell can evade infection via a disruptive mutation in the gene for the receptor, but such mutations also abolish the native function of the receptor [3]. A solution for the cell might be a specific missense mutation that disrupts interaction with the pathogen while maintaining native function, but mutations of this type are typically much rarer than general loss-of-function mutations [4].

Phage λ is one of many phages that bind to a nutrient transporter, recognizing and infecting through the maltose-specific porin LamB on the *E. coli* outer membrane [1, 2, 5–7]. When *E. coli* is cultured with *λ* in rich media, mutations accrue in the maltose regulon, most commonly large and/or frameshifting deletions in the *lamB* gene or its transcriptional activator *malT* [8, 9]. LamB is a trimeric β-barrel pore that spans the outer membrane of *E. coli* and facilitates the diffusion of maltose and maltose-derived oligosaccharides like maltodextrin into the periplasm. Loss-of-function mutations in *lamB* lead to cells that are incapable of transporting these nutrients, therefore incurring a cost in some environments, although rare missense mutations have been identified in *lamB* that confer *λ* resistance and maintain the ability of LamB to transport maltose [8, 10, 11].

The structure of LamB consists of 18 transmembrane *β*-sheets, each linked to the adjacent sheets by a short linker on the periplasmic face and a long or short loop on the extracellular face [12]. The extracellular loops are the most rapidly evolving portions of the protein and play a dual role in specifying the transported substrates and forming binding sites for proteins of pathogens, including *λ* [10, 13, 14]. In particular, the extracellular loops L4, L5, L6 and L9 are highly diverged within Enterobacteriaceae (Fig. S1, available in the online version of this article), likely due to selective pressure to avoid phages. Most variation within these loops is missense, as opposed to indels or structural variation, suggesting that missense mutations drive much of the long-term evolution of this protein.

In this study, we set out to explore the space of possible missense mutations in *lamB* and how they affect both maltodextrin transport and *λ* infectivity. By scoring both activities, we observe global correlation between *λ*-sensitivity and maltodextrin transport, suggesting that most mutations act through a mechanism like destabilization that affects all LamB activities. Mutations that affect the two activities occurred throughout the LamB structure. At a finer scale, individual mutations that specifically affect a single activity could be identified, and they strongly implicate loop L6 in determining *λ*-sensitivity.

## Methods

### Strategy

We adopted a strategy known as ‘deep mutational scanning’ [15], wherein, typically, cells carrying a library of genetic variants in a gene are subjected to a selection condition that requires a function of that gene, and mutational effects are estimated from the frequency changes of each variant as determined by deep sequencing. We generated a library of variants in *lamB* using error-prone PCR, cloned the library into a *lac*-inducible expression vector, and expressed the variants in an *E. coli* strain knocked out for *lamB*. Cells were then selected separately either for their ability to grow in rich media containing *λ*, or in minimal media with maltodextrin as a sole carbon source. The *lamB* gene was amplified from cells after the selection conditions and control conditions, and mutation frequency was estimated by short-read sequencing of random fragments of the *lamB* amplicon. We calculated functional scores based on differences in the frequency of each mutation in the selection condition compared to the corresponding control condition and used score thresholds to categorize mutational effects. For some mutations categorized as *λ*-resistant and Mal^+^, we assayed the effect of the individual mutation in an otherwise wild-type *lamB* context.

### Strains

All assays were done in a DH10B background. DH10B(*lamB* ^∆^) was generated using the *λ*-red system. Briefly, pSIM5 [16] was transformed into DH10B and was induced by heat shock at 42 °C. A linear cassette containing the genes *tetA* and *sacB* flanked by homology to the *lamB* locus was then transformed in, and recombinants were selected by tetracycline. After verifying the *lamB* deletion, the cassette was removed by transforming an oligonucleotide that annealed flanking the cassette, and selecting on fusaric acid and sucrose. The deletion of the *lamB* coding sequence was verified by Sanger sequencing and the strain was cured of pSIM5 by growing at 37 °C and choosing a chloramphenicol-sensitive colony.

*λ*_sam7_ was generated by packaging *λ* DNA from NEB (N3011, cI857 ind1 Sam7) into *λ* particles using the MaxPlax *λ* packaging extract from Lucigen (MP5120). Prior to the assay, ~10^5^ packaged phages were plated on host-strain LE392MP (Lucigen SS000437-D), grown for 5 h at 37 °C, and washed into TMS (10 mM Tris-HCl+10 mM MgSO_4_+100 mM NaCl). The lysate was cleared by centrifugation and filtered through a 0.2 µm filter, and had a titre of 3.2×10^8^ p.f.u. ml^–1^. The *λ*_sam7_ is amber suppressible for lysis, such that it will not lyse cells that recognize an amber codon as a stop codon. Additionally, *λ*_sam7_ contains the temperature-sensitive cI857 allele, such that it is obligately lytic at 37 °C, but can lysogenize at 30 °C. In all cases in this paper, infection with *λ*_sam7_ was carried out at 37 °C.

*λ*_JL801_ was a gift from Ben Kerr, and a plate lysate was generated via the same method as for *λ*_sam7_, except DH10B was used as the host. The titre was 9.3×10^8^ p.f.u. ml^–1^. *λ*_JL801_ differs from *λ*_sam7_ in that it is not amber suppressible, and the cI gene contains a large deletion in the 5′ end, rendering the phage obligately lytic.

### Error-prone PCR and library generation

The *lamB* gene was amplified from DH10B using PCR and cloned into the *lac*-inducible high-copy expression vector p44K (addgene 45800) using Gibson assembly [17] to produce p44K-lamB. A miniprep of this plasmid was used as the template for error-prone PCR with the Agilent GeneMorph II kit, using 800 ng or 2400 ng of plasmid per reaction. PCR products were treated with DpnI to remove template plasmid, cleaned up on a Zymogen DNA Clean and Concentrator column, and cloned back into the plasmid backbone using Gibson Assembly. The assembled plasmid was transformed into DH10B, obtaining an estimated 1.1×10^6^ transformants per library. The plasmid library was miniprepped and transformed back into DH10B(*lamB* ^∆^).

### Media and culture conditions

For selections, 200 µl stock of the library was inoculated into 5 ml LB and shaken for 1 h at 37 °C to recover. Carbenicillin was then added to 50 µg ml^−1^ and IPTG to 100 µM, and the culture was shaken for 1 additional h, or until the culture reached OD_600_=0.4. For each biological replicate, 1 ml cells were then spun down (30 s × 12 700 r.p.m.) and resuspended in 200 µl TMS.

For the *λ*-resistance selection, 20 μL of the cells were diluted into 10 ml LB+0.2 % maltose+10 mM MgSO_4_+100 µM IPTG+50 µg ml^−1^ carbenicillin for the control condition, or the same buffer with 10^6^ p.f.u. ml^–1^ *λ*_sam7_ for the selection condition. The cultures were shaken for 16 h at 37 °C, then collected.

For the maltodextrin selection, 20 μL of the cells were diluted into M9+0.4 % glucose+80 µg ml^−1^ leucine+100 µM IPTG+50 µg ml^−1^ carbenicillin for the control condition, or the same media with 0.4 % maltodextrin and no glucose for the selection condition. The cultures were shaken for 36 h at 37 °C, then collected.

### Sequencing library preparation

After the plasmids were miniprepped from the selections, the *lamB* coding sequence and flanking ~100 bp were amplified using qPCR for 11 cycles, and the template was digested with DpnI. The amplicon was then subjected to tagmentation using the Illumina Nextera kit. The fragments were amplified and indexed by a further five cycles of PCR, then prepared for Illumina sequencing.

### Sequencing and sequence processing

The Nextera fragments were sequenced on the Illumina NextSeq, obtaining 2–4 million reads per replicate using 2×38 bp reads, or 1–2 million reads per replicate using 2×75 bp reads. The raw read pairs were collapsed into unique read pairs, and unique read pairs represented by less than five raw reads were discarded. Trim Galore was used to remove the Nextera adaptor sequences and Pear was used to merge read pairs that overlapped at the 3′ end. The unique reads were then aligned to the wild-type *lamB* amplicon using Bowtie2. Samtools and pysamstats were used to count the matches and mismatches between the aligned unique reads and the template amplicon, and the counts were all given an additional pseudocount of 0.1.

### Calculating functional scores

For each mutation with an average of five or more counts in the libraries from the control conditions, we calculated enrichments, and converted those to functional scores. For the *λ* selection, we calculated the enrichments, *E*_*λ*_ as follows:

$$E_{\lambda }=\log_{2}\left[\left(C_{\lambda ,mut}/C_{\lambda ,wt}\right)/\left(C_{LB,mut}/C_{LB,wt}\right)\right]$$

where *C* is a count of how many times a base appeared at a given position, *λ* indicates the count from the LB+*λ* selected population, LB indicates the count from the control population, mut indicates the count refers to a mutant base, and wt indicates the count of the reference base at that position.

Enrichments were converted to functional scores by linearly scaling them such that the median synonymous score was set to 1 and the median nonsense score was set to 0.

$$F_{\lambda }=\frac{E_{\lambda \;-\;median}\left(E_{\lambda ,stop}\right)}{median\left(E_{\lambda ,syn}\right)-median\left(E_{\lambda ,stop}\right)}$$

where *E_λ_* is the enrichment calculated above,syn refers to enrichments for all mutations that are synonymous to the wild-type sequence, and stop indicates that the mutation is a nonsense mutation.

Similarly, for the maltodextrin selection, we calculated

$$E_{malt}=\log_{2}\left[\left(C_{M,mut}/C_{M,wt}\right)/\left(C_{G,mut}/C_{G,wt}\right)\right]$$

where *M* indicates the count from the M9+maltodextrin population and *G* indicates the count from the M9+glucose population.

Enrichments were converted to functional scores as follows:

$$F_{malt}=\frac{E_{malt\;-\;median}\left(E_{malt,syn}\right)}{median\left(E_{malt,stop}\right)-median\left(E_{malt,syn}\right)}$$

### Site-directed mutagenesis for individual mutations

For 20 mutations that were called *λ* ^r^Mal**^+^**, we generated variants containing only the single mutations. Primers containing the mutation with 20 bp of the wild-type sequence on either end were ordered along with reverse primers abutting the 5′ end. These were used to amplify the entire p44k-lamB plasmid, and the PCR product was treated with DpnI to remove template and T4 polynucleotide kinase to phosphorylate the ends, then ligated to form a circular product. The ligation products were transformed into DH5α and the *lamB* coding sequence was Sanger sequenced to confirm the directed mutation and the absence of other mutations. The sequence-verified plasmids were transformed back into DH10B(*lamB* ^∆^).

### Growth curves for individual mutations

For each of the individual mutations, cultures were grown overnight by inoculating a single colony into 2 ml LB ampicillin and shaking at 37 °C. In the morning, the cultures were back-diluted 1 : 50 into LB+ampicillin+100 µM IPTG and grown for 2 h. The cells were harvested and resuspended into an equal volume of TMS. The cells were then diluted in duplicate (10 µl cells+190 µl media) onto a 96-well plate, where the media was LB+0.2 % maltose+10 mM MgSO_4_+100 µM IPTG+10^4^ p.f.u. ml *λ*_JL801_ when measuring *λ* resistance, or M9+100 µM IPTG+0.4 % maltodextrin+80 µg ml^−1^ leucine when measuring maltodextrin transport. The plate was incubated at 37 °C with shaking, and OD_600_ was measured every 10 min for 48 h.

## Results

### A sequencing-based approach to map the effects of mutation on *lamB*

**Fig. 1.**
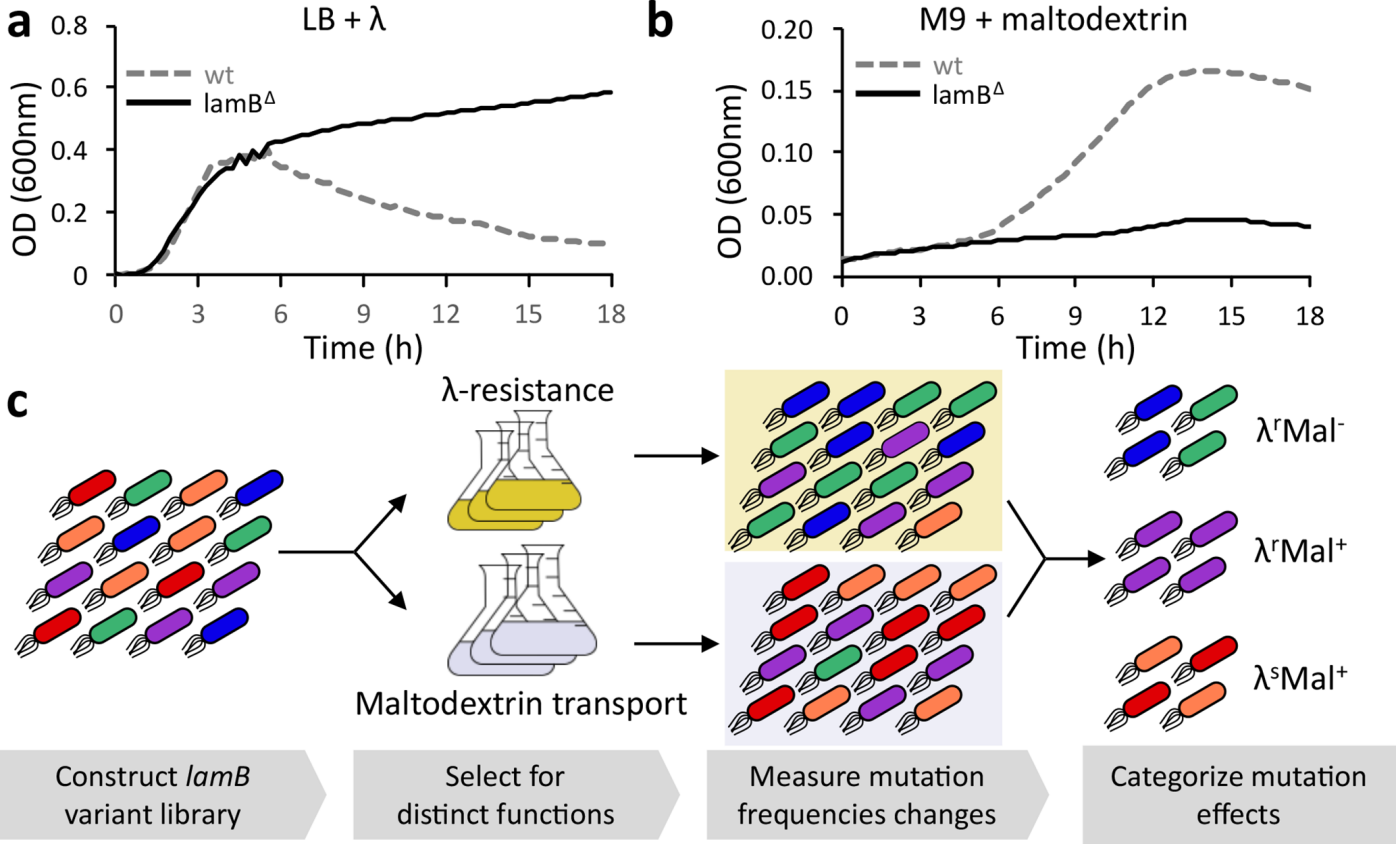
Separation of the functions of LamB via growth in different selective conditions. (a) *E. coli* (strain DH10B) containing wild-type *lamB* or a deletion of the entire *lamB* coding sequence were grown for 18 h in LB+*λ*_JL801_ at 37 °C, and OD_600_ was measured every 10 min. The average of three replicate wells are plotted over time. (b) *E. coli* (strain DH10B) containing wild-type *lamB* or a deletion of the entire *lamB* coding sequence were grown for 18 h in M9+maltodextrin at 37 °C, and OD_600_ was measured every 10 min. The average of three replicate wells are plotted over time. (c) The experimental strategy for determining effects of missense mutations involves subjecting a mutational library of *lamB* variants, each expressed from a plasmid in an *E. coli* cell, to distinct selection pressures of *λ* resistance or maltodextrin transport. This general strategy is sometimes called deep mutational scanning [15].

We first sought to determine the fitness consequences for *E. coli* of *lamB* expression under different selective conditions. Deletion of *lamB* confers a large fitness advantage in LB media containing *λ* (Fig. 1a), but a large cost in M9+maltodextrin, media in which maltose-derived oligosaccharides are the sole carbon source (Fig. 1b). We therefore decided to use these conditions to categorize variants with respect to *λ*-sensitivity and maltodextrin transport using a pooled assay, in which variants compete for growth either in the presence of *λ* (in rich media) or in maltodextrin as the sole carbon source, with the frequency of each mutation in selected and control conditions determined by high throughput DNA sequencing (Fig. 1c) [15, 18].

To generate variants for pooled assays, we produced two libraries, each consisting of greater than 1 million variants of *lamB*, by amplifying the 1341 bp coding sequence of *lamB* with error-prone PCR with different amounts of starting template (see Methods). The libraries had per-base error rates of 0.52 and 0.063 %, as determined by comparing the number of mismatches in successfully aligned fragments to the number of matched aligned bases in the control libraries. Initial tests on 11 random variants from each library showed only moderate loss of *λ*-sensitivity (6/11 from each library retained sensitivity), indicating that even with a high mutational burden, a large fraction of variants produced functional protein (Fig. S2). Because both libraries had many *λ*-sensitive variants, we used the higher mutation rate library (7 mutations/variant) in the *λ* selection. However, we elected to use the lower mutation rate library (0.84 mutations/variant) for the maltodextrin selection to get a better estimate of the effects of single mutations.

We induced expression of the plasmid-borne variant libraries by IPTG in the *lamB*-deficient strain DH10B(*lamB* ^∆^), and diluted the cultures 1 : 100 into control media (LB for the *λ* selection or M9+glucose for the maltodextrin selection) and selective media (LB+*λ* for the *λ* selection or M9+maltodextrin for the maltodextrin selection) in biological triplicate. In the *λ* selection, we used the *sam7* strain of *λ*, which carries a lysis gene interrupted by an amber stop codon. Because this strain infects susceptible cells without releasing *λ* progeny into the culture, it prevents the phage population from dramatically increasing over the course of the experiment, and *λ* cannot evolve adaptations to the variation in *lamB*. After overnight growth of the cultures, we isolated plasmids from each and amplified the *lamB* coding sequence and ~200 bp flanking sequence with 12 cycles of PCR.

Rather than attempting to measure the frequency of each full-length *lamB* variant, we instead measured the frequency of each mutation, disregarding other co-occurring mutations. We assume that the average function of variants containing a given mutation, compared to variants not containing it, will reflect the functional effect of that mutation on the wild-type protein [19, 20]. To correct for sequencing errors, we developed a strategy of randomly fragmenting the gene with Tn5 and using the start site, stop site, and mutations of each fragment as an identifier by which to construct a consensus sequence, using a minimum count of five reads to call a fragment. For each library, we generated ~50 000 fragments, with a median fragment length of 63 bp (Fig. S3a). The number of sequenced fragments was several orders of magnitude less than the ~10^8^ possible fragments, such that each unique fragment should represent a single transposition event. For each base in the sequence, we counted the number of fragments containing the mutant base and the wild-type base. We calculated an enrichment score for each mutation as the log_2_ ratio of wild-type-normalized counts between the LB+*λ* and LB conditions or the M9+maltodextrin and M9+glucose conditions (see Methods).

### *lamB* missense mutations that confer *λ*-resistance

**Fig. 2.**
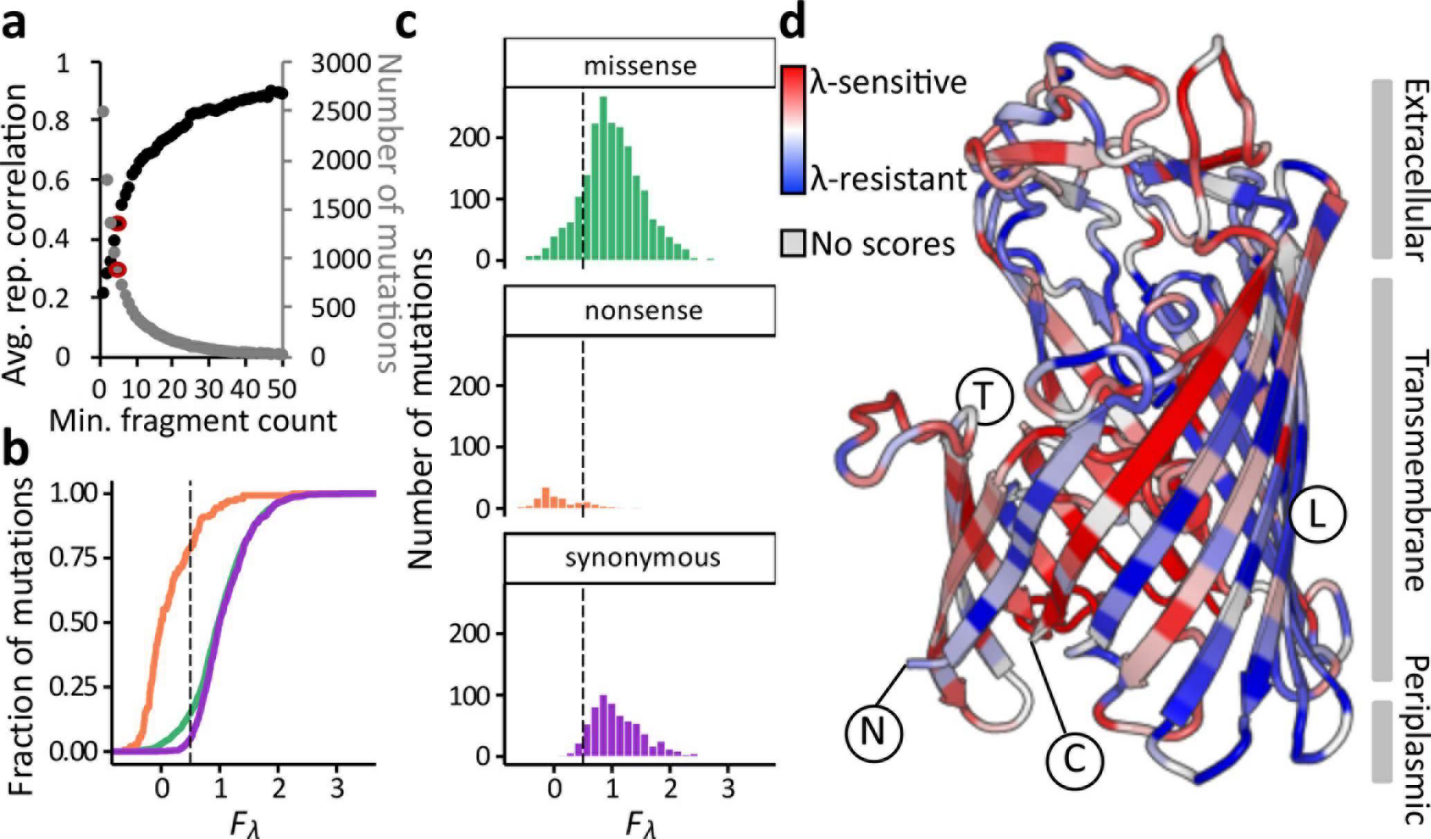
Missense mutations can drive large changes in *λ* infectivity. (a) Mutations in *lamB* were scored in six replicates for their effects on *λ* sensitivity by comparing mutation frequencies before and after selection in LB+*λ*, and the scores were compared between replicates. The correlation between replicates is a function of the fragment input count threshold, and increases as the threshold is raised. However, raising the fragment input count threshold decreases the number of mutations that can be analysed because fewer meet the threshold. The fragment input count threshold used throughout the manuscript, five, is highlighted in red. ‘rep’: replicate. (b) The empirical cumulative distribution function of *F*_*λ*_ scores, which estimate mutational effects on *λ*-sensitivity from zero (nonsense-like) to one (synonymous-like). The missense distribution mostly tracks the synonymous distribution, with a slightly longer tail to the left. Green: missense mutations. Orange: nonsense mutations. Purple: synonymous mutations. The dotted line represents the threshold, *F*_*λ*_=0.5, above which mutations are initially called *λ*-sensitive. The placement of this line is reconsidered on the basis of individual assays for Figs 4 and 5. (c) The distribution of fitness effects for the *λ* selection, for all scored single mutations, averaged over six replicates. Colours and dotted line are as in (b). (d) The mean *F*_*λ*_ score of mutations at each residue in LamB, coloured relative to other residues from *λ*-resistant (red) to *λ*-sensitive (blue). Structural features described in the text are annotated, but see also Fig. S4. N: the amino-terminus of the mature protein. C: the carboxyl-terminus of the mature protein. T: the face of the protein that binds other monomers to form the functional trimer. L: the face of the protein exposed to the lipid bilayer.

We estimated a function score for *λ* sensitivity (*F*_*λ*_) by scaling the enrichment scores from the *λ* selection such that the median nonsense mutation, expected to be *λ* **^r^**, was assigned a score of 0 and the median synonymous mutation, expected to be *λ* **^s^**, was assigned a score of 1. Note that this scaling reverses the axis such that enriched variants (with resistance to *λ*) have lower scores. We ignored mutations represented by fewer than five fragments in the control condition. This fragment cutoff was based on the number of mutations that passed the cutoff and the concordance between replicates (Fig. 2a). At this threshold, replicates were moderately correlated, with a mean Pearson’s *r*=0.443 between biological replicates and *r*=0.481 between technical replicates. The relatively modest correlation reflects the scores as being averaged over many different variants containing each mutation, and that these variants tended to be different in each replicate due to the large size of the library. To reduce and quantify error, we averaged scores over six replicates, consisting of three biological replicates each performed in two technical replicates.

As expected, nonsense mutations had low *F*_*λ*_ scores compared to synonymous or missense mutations (Fig. 2b, c). By comparison, synonymous mutations scored relatively well and did not overlap much with the nonsense distribution. Among missense mutations, there was a wide distribution of scores. Over most of the range, the missense mutations were similarly distributed to the synonymous mutations. However, the missense distribution had a longer tail to the left (Fig. 2c). To classify missense mutations as ‘*λ*-sensitive,’ we set a threshold at *F*_*λ*_=0.5, the midpoint between the median nonsense (*F*_*λ*_=0) and median synonymous scores (*F*_*λ*_=1) (Fig. 2c). A total of 302 missense mutations (15%) fell below this threshold, along with 79 % of nonsense variants and 5 % of synonymous mutations.

Spatially, the most *λ*-resistant and the most *λ*-sensitive mutations fell throughout the protein structure, including in both the transmembrane *β*-sheets and the extracellular loops (Fig. 2d). Stop codons were deleterious (i.e. conferring *λ*-resistance) at any point in the coding sequence, even the last few residues. Most of the trans-membrane strands facing against the lipid bilayer primarily contained *λ*-resistant mutations, with the exception of the C-terminal strand, which contained many *λ*-sensitive mutations (Fig. S4). By contrast, most of the trans-membrane strands facing the other monomers contained an abundance of *λ*-sensitive mutations, with the exception of the N-terminal strand (Fig. S4). The relative intolerance to mutation of the N-terminal strand compared to the C-terminal strand, despite their physical proximity, suggests that N-terminal mutations may be disrupting insertion of the protein into the membrane, which occurs in the N-to-C direction [21], rather than protein stability *per se*. However, this hypothesis fails to explain why mutations at the trimer interface were relatively well-tolerated to maintain *λ*-sensitivity. LamB functions only as a trimer, and we hypothesize that it trimerizes with sufficient affinity that most single mutations would fail to disrupt assembly. This result is consistent with the extreme stability of LamB trimers, which withstand temperatures up to 70 °C in 9M urea [22].

### Most *lamB* mutations do not disrupt maltodextrin transport

**Fig. 3.**
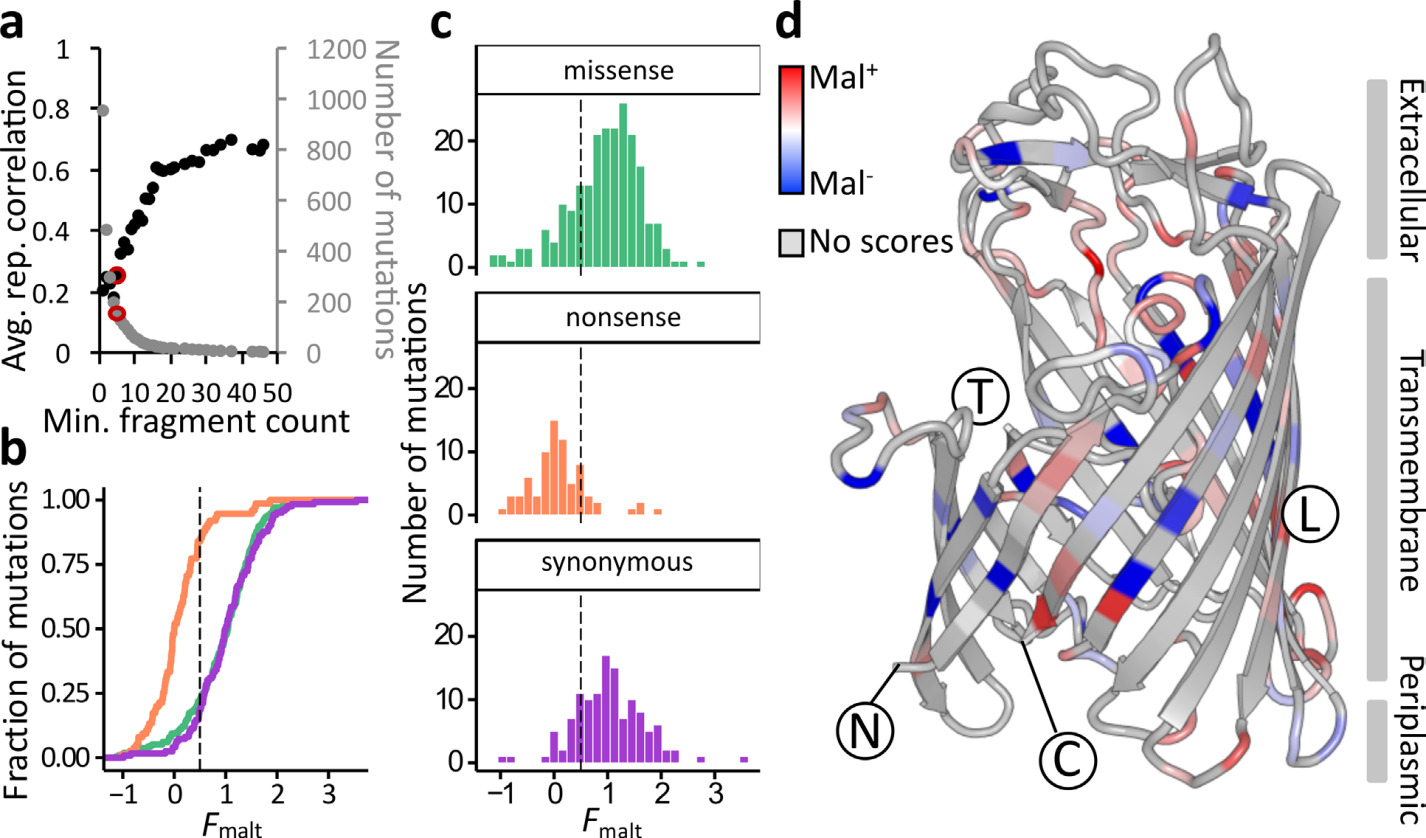
Most missense mutations are unable to strongly disrupt maltodextrin transport. (a) Mutations in *lamB* were scored in six replicates for their effects on maltodextrin transport by comparing mutation frequencies before and after selection in M9+maltodextrin, and the scores were compared between replicates. The relationship between the replicate correlation and fragment input count threshold is as for Fig. 2. The fragment input count threshold of five is highlighted in red. ‘rep’: replicate. (b) The empirical cumulative distribution function of *F*_malt_ scores, which estimate mutational effects on maltodextrin transport from zero (nonsense-like) to one (synonymous-like). The missense distribution is not significantly different from the synonymous distribution (*P*=0.65, Kolmogorov–Smirnov test). Green: missense mutations. Orange: nonsense mutations. Purple: synonymous mutations. The dotted line represents the threshold, *F*_malt_=0.5, above which mutations are called Mal^+^. (c) The distribution of fitness effects for the maltodextrin selection, for all scored single nucleotide mutations, averaged over six replicates. Colours and dotted line are as in (b). (d) The mean *F*_*λ*_ score of mutations at each residue in LamB, coloured relative to other residues from Mal^+^ (red) to Mal^-^ (blue). Structural features described in the text are annotated, but see also Fig. S4. N: the amino-terminus of the mature protein. C: the carboxyl-terminus of the mature protein. T: the face of the protein that binds other monomers to form the functional trimer. L: the face of the protein exposed to the lipid bilayer.

We next investigated the fitness effects of *lamB* mutations on the transport of maltodextrin. We estimated functional maltodextrin transport (*F*_malt_) by scaling the enrichment scores such that the median nonsense mutation, expected to be Mal**^-^**, was assigned a score of 0, and the median synonymous mutation, expected to be Mal**^+^**, was assigned a score of 1. Because of the overall lower mutation rate in the library used for this selection (each *lamB* variant on average contained 0.84 mutations), we captured fewer mutations overall, with lower fragment counts and higher variance. Replicates correlated only modestly (*r*=0.26), and thus to reduce error, we again averaged scores over six replicates, consisting of three biological replicates each performed in two technical replicates (Fig. 3a). Regardless, we were able to score hundreds of mutations for their effect on the transport of maltodextrin.

We observed a separation of the nonsense and synonymous mutations, with the missense mutations spanning the range of both distributions (Fig. 3b). We set a threshold for assigning mutations as Mal**^+^**or Mal**^-^** midway between the median nonsense (*F*_malt_=0) and median synonymous mutation (*F*_malt_=1), at *F*_malt_=0.5. At this threshold, 166 missense mutations (78%) fell above this threshold and were called Mal^+^, along with 81 % of synonymous mutations and 16 % of nonsense mutations (Fig. 3c). Unlike in the selection for *λ* resistance, in the selection for growth on maltodextrin, the distributions of missense and synonymous mutations were not statistically significantly different from each other (*P*=0.65, Kolmogorov–Smirnov test) (Fig. 3b), implying that most *lamB* missense mutations are not highly disruptive to maltodextrin transport. The nonsense distribution was significantly different from both the missense and synonymous distributions (*P*<10^−6^, Kolmogorov–Smirnov test). We conclude that most single amino acid substitutions in *lamB* do not destroy the ability of the protein to transport maltose derivatives, in contrast to the observation that many mutations in the transmembrane sheets confer at least partial *λ*-resistance. This finding can be explained if sensitivity to *λ* and maltose transport differ in the amount of LamB protein needed to confer each activity, or if *lamB* is sensitized to the effects of mutations by the higher overall mutation rate in the *λ* selection. High- and low-scoring mutations fell in all regions of the protein, with no region appearing broadly tolerant of mutations (Fig. 3d).

### Comparison of the sequence–function maps for resistance to *λ* and maltodextrin transport

**Fig. 4.**
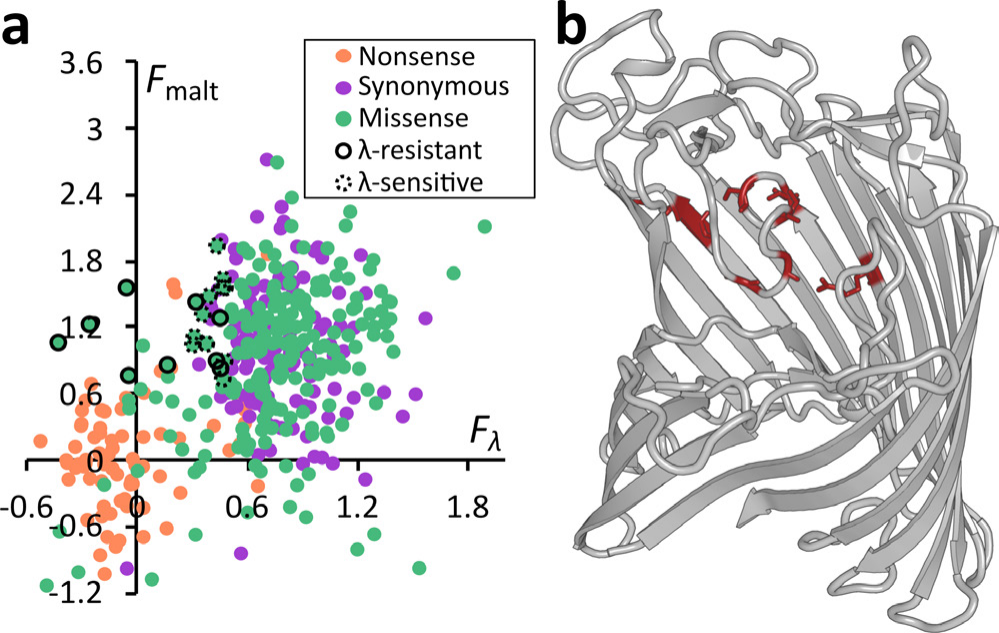
*λ* ^r^Mal^+^ mutations are relatively rare and constrained to loop L6. (a) The functional scores, *F*_malt_ and *F*_*λ*_, for each variant are significantly correlated between selections, with Pearson’s *r*=0.495, *P*<10^−6^. Mutations that were chosen for individual assays are outlined, with solid outlines indicating agreement between the individual and the pooled assay, while dotted outlines indicate the mutations that are at least partially sensitive to *λ* in isolation. Green: missense mutations. Orange: nonsense mutations. Purple: synonymous mutations. (b) The mutations that were determined in individual assays to be *λ* ^r^Mal^+^ are shown in red on the structure of LamB. Eight of these are either contained on or directly contact loop L6, with the ninth occurring on the signal peptide (not shown).

Scores for *λ*-sensitivity and maltodextrin transport were correlated (*r*=0.495, *P*<10^−6^), and plotting mutations on both axes separates *λ* ^s^Mal^+^ synonymous mutations from *λ* ^r^Mal^-^ nonsense mutations (Fig. 4a). Simply asking whether (*F*_malt_ +*F*_*λ*_)/2>0.5 is sufficient to correctly classify 92 % of these mutations as nonsense or synonymous mutations, which is higher accuracy than using either selection in isolation. Among missense mutations, the overall correlation of *λ*-sensitivity and transport of maltodextrin implies that most mutations affect a common protein property, such as stability or trafficking, that is important for both phenotypes. At a finer scale, individual mutations can be identified that appear to specifically interfere with only one activity. However, the identification of these mutations necessarily depends on the score thresholds used to categorize mutations.

In the previous analyses, we used thresholds at *F*=0.5 to phenotypically categorize mutations. In the case of the maltodextrin selection, this threshold is probably close to optimal, as fitting Gaussian distributions to the synonymous and nonsense distributions yields an intersection at *F*=0.51 that separates the functional and non-functional distributions. In the case of *λ*-sensitivity, it is more difficult to determine the most appropriate threshold because the score distributions are non-Gaussian and because many missense mutations fall in an intermediate range between the synonymous and nonsense ranges. We therefore decided to take an empirical approach to refine our estimate of the appropriate *F*_*λ*_ threshold. We individually assayed 20 putatively *λ* ^r^Mal^+^ mutations with *F*_*λ*_ scores ranging from −0.5 to 0.5. We cloned each of these mutations back into the parent plasmid by site-directed mutagenesis (see Methods), and measured growth curves for each resulting transformed strain in M9+maltodextrin and LB+*λ*. Of the 20 variants, all of them grew in M9+maltodextrin, although several had either a delay in reaching log-phase, or a decreased maximum growth rate, or both (Fig. S5). Nine were fully *λ*-resistant, ten were *λ*-sensitive and one was of intermediate resistance. The mutations that were shown to be individually *λ*-sensitive were close, in the pooled assay, to the threshold of *F*_*λ*_=0.5, with the lowest *F*_*λ*_=0.312 (Fig. 4a). By contrast, the *λ*-resistant mutations had an average *F*_*λ*_=0.124 (Fig. 4a). From these results, we conclude that decreasing the threshold to *F*_*λ*_≈0.3–0.35 effectively removes moderately scoring mutations that do not confer full resistance to *λ*, although mutations above the threshold may confer partial resistance in the context of a pooled assay and/or other mutations.

### Mutations conferring *λ* ^r^Mal^+^ phenotype are restricted to loop L6

Of the nine individually assayed mutations sufficient to confer *λ* ^r^Mal^+^, five occur in the extracellular loop L6, and three others in residues that directly contact this loop (Fig. 4b). The concentration of *λ* ^r^Mal^+^ mutations in this loop strongly implicates loop L6 as a key structural determinant of *λ* binding. While *λ*-resistance mutations in loop L6 have been previously reported [10, 23], the loop structural feature has not been described as a hotspot for *λ*-resistance and is less surface-exposed than many of the other extracellular loops [23]. The ninth individually assayed *λ* ^r^Mal^+^ mutation falls in the signal peptide and is not part of the mature protein. We hypothesize that reducing LamB protein levels by altering the signal peptide, without completely abrogating expression, confers some level of *λ*-resistance. The remaining 11 mutations appeared to confer *λ* ^r^Mal^+^ in the pooled selection, but were not sufficient to do so in individual assays. These mutations were more spatially scattered, but include changes to residues that face the inside of the lipid bilayer, for instance, and might be expected to affect protein stability or abundance, but not *λ*-binding directly (Fig. S6). That these mutations exist but have comparatively moderate *F*_*λ*_ scores (Fig. 4a) suggests that the stability or abundance effects of mutations are unlikely to act as the primary drivers of *λ*-resistance in a population with many alleles of *lamB*.

### Among *λ* ^r^ mutations, Mal^+^ and Mal^-^ phenotypes are approximately equally likely

**Fig. 5.**
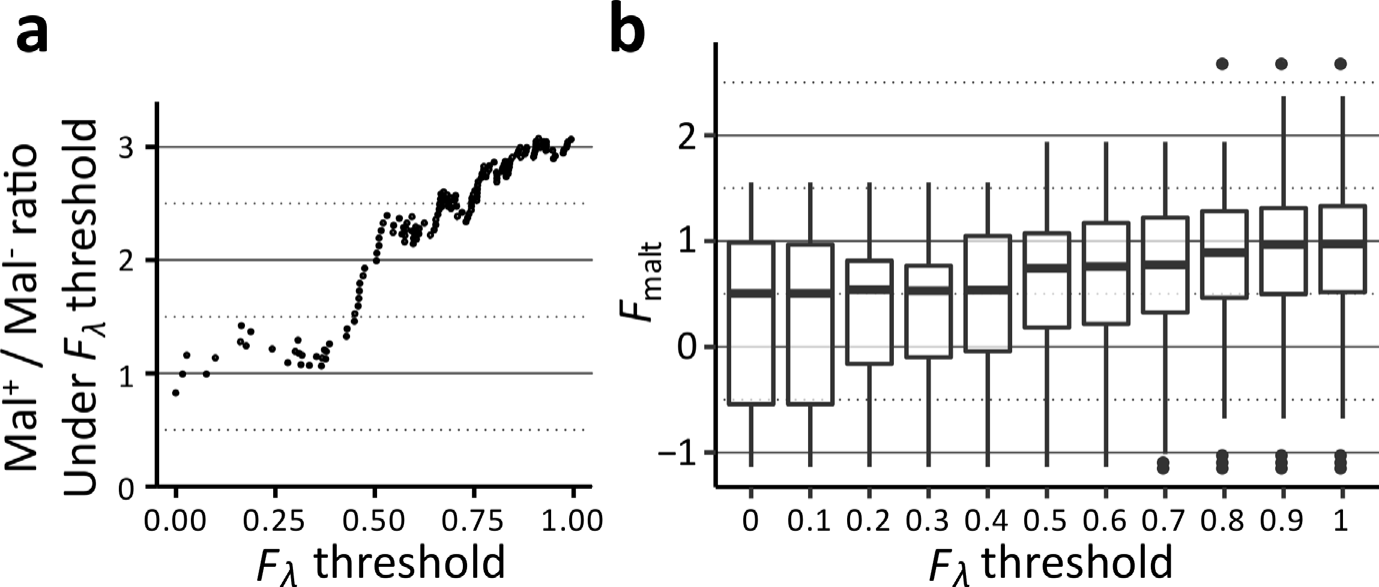
Comparison of Mal^+^ and Mal^-^ mutations across a range of *F_λ_* thresholds. (a) For *λ*-resistant variants, as determined by *F*_*λ*_ below a defined threshold, we calculate the ratio of mutations that are Mal^+^ or Mal^-^. As the threshold is decreased below *F*_*λ*_=0.35, this ratio approaches one, although noise increases due to the lower number of mutations under the more stringent threshold. The *F*_malt_ threshold used for this and all other analyses is 0.5. (b) The distributions of *F*_malt_ scores below a given threshold of *F*_*λ*_ are shown. The median *F*_malt_ decreases from ~1.0 (equivalent to a synonymous codon) with a high threshold to ~0.5 with a lower threshold at *F*_*λ*_=0.4. However, the median *F*_malt_ score does not appreciably change if the threshold is decreased further.

To predict how the sequence–function map of *λ*-sensitivity might affect how *E. coli* acquires resistance to *λ*, we asked whether *λ* ^r^ missense mutations are more likely to confer a Mal^+^ or Mal^-^ phenotype. Because our individual assays suggested that the appropriate *F*_*λ*_ threshold for classifying *λ* ^r^ mutations is likely lower than *F*_*λ*_=0.5, closer to *F*_*λ*_≈0.3–0.35, we decided to compare Mal^+^ and Mal^-^ mutations across a broad range of *F*_*λ*_ thresholds, rather than picking a single threshold. Below a threshold of *F*_*λ*_=0.4, the ratio of Mal^+^ to Mal^-^ approaches 1.0 (Fig. 5a), implying that a randomly chosen *λ* ^r^ mutation is approximately equally likely to be Mal^+^ or Mal^-^. This effect can also be seen by looking at the distribution of *F*_malt_ scores. If *F*_*λ*_<1, the median *F*_malt_ is ~1.0 (comparable to a synonymous mutation), but if *F*_*λ*_<0.4, the median *F*_malt_ drops to ~0.5 (Fig. 5b). The median *F*_malt_ score does not change appreciably between *F*_*λ*_=0.0 and *F*_*λ*_=0.4. We interpret these data as implying that when *λ*-resistance is mediated by a randomly chosen missense mutation, this mutation is approximately equally likely to disrupt or maintain maltodextrin transport. This result seems surprising given that there are many more ways to disrupt the structure of a protein compared to specifically disrupting a single binding site for a pathogen. However, the extreme stability of folded LamB may reduce the number of mutations that prevent correct folding, thereby resulting in many *λ*-resistance mutations that still allow maltodextrin transport [18, 22, 24].

## Discussion

By carrying out assays of LamB variants for their ability to promote two activities – infection by λ and transport of maltodextrin – that inherently conflict, we establish independent sequence-function maps for this single protein. Most missense mutations have similar effects on both activities, pointing to the primacy of fundamental protein properties, like stability, on the effects of mutations. Despite the overall agreement of mutational effects between the two selections, mutations that specifically disrupt a single function, *λ*-sensitivity, could be isolated and were highly spatially clustered.

These results are subject to certain caveats. First, the mutational complexity of the libraries differed between the two selections; second, the strength of selection may have differed between selections; and third, mutations were scored by comparing heterogeneous variants that do and do not contain each mutation rather than directly assessing a single mutation in a wild-type background. These caveats are consistent with some amount of noise in the data, such as the fact that only 92 % of synonymous and nonsense mutations can be correctly classified as such using their *F*_*λ*_ and *F*_malt_ scores, and that only 9/20 mutations putatively called as *λ*-resistant were sufficient to confer complete *λ*-resistance in individual assays. While all technical strategies to measure phenotypes have limitations, we observed a clear separation by function in both selections, demonstrating that the overall distribution of fitness effects is meaningful. Furthermore, by individually assaying mutations, we were able to refine our set of *λ* ^r^Mal^+^ mutations, improving our estimates of the appropriate *F*_*λ*_ threshold for determining resistance and helping us narrow in on loop L6 as the major structural determinant of *λ* binding.

Nearly all of the missense mutations that conferred complete *λ*-resistance in individual assays are involved in a single structural motif, loop L6. Co-crystallization of LamB with maltose suggests that maltose binding is mostly coordinated by loop L1 and loop L3, along with the inward faces of some transmembrane beta-strands [12, 13]. This spatial segregation likely explains why some *lamB* mutations can disrupt *λ* binding while maintaining maltodextrin transport. Given that *λ* ^r^Mal^+^ mutations are relatively rare and involve only a single loop of the 18-loop LamB, it might be expected that most *λ*-resistance mutations would be general loss-of-function (i.e. conferring the *λ* ^r^Mal^-^ phenotype). However, we find that approximately half of the missense mutations that led to *λ*-resistance did not eliminate maltodextrin transport. We therefore expect that in an environment containing both *λ* and maltodextrin, *E. coli* should be able to directly acquire *λ*-resistance in a single step, without sacrificing maltose transport. An alternative two-step pathway would be for *E. coli* to first acquire *λ*-resistance via a general loss-of-function mutation (*λ* ^r^Mal^-^) followed by a compensatory mutation that reverts the phenotype to *λ* ^s^Mal^+^ once *λ* has left the environment. Although the single-step pathway seems more advantageous to the bacteria, it can precipitate an evolutionary arms race if *λ* acquires counter-resistance, driving either *E. coli* or *λ* to extinction [25–30]. In experimental evolution studies that compete *λ* with *E. coli *, *λ* is typically the first to go extinct, yet we know that phage populations and species can persist for decades in the wild [25–27, 31, 32]. When species are known to coevolve stably for long periods of time, a different set of dynamics, called fluctuating selection dynamics, are often presumed to dominate [33, 34]. Fluctuating selection dynamics would be more consistent with the two-step pathway, with *E. coli* periodically fluctuating between *λ* ^r^ and *λ* ^s^ phenotypes, but such a model would incorrectly predict that *λ* ^r^Mal^+^ mutations should be difficult to acquire relative to general loss-of-function mutations.

To square our results with these evolutionary considerations, we hypothesize that selection for maltose transport is close to zero in environments where *λ* and *E. coli* interact [35, 36]. This hypothesis predicts that the relative abundance of *λ* ^r^Mal^+^ and *λ* ^r^Mal^-^ mutations should be equal rather than weighted toward *λ* ^r^Mal^-^. Furthermore, phages are thought to cause more selection in nutrient-rich conditions like the human gut, where maltose is not a preferred carbon source [35]. The maintenance of the Mal^+^ phenotype could thus be driven by exposure to periodic nutrient-poor conditions when phages are mostly absent and fluctuating selection dynamics are thought to be favoured [35, 37, 38].

A prediction that arises from this work is that a receptor whose loss is tied to a more significant fitness defect in phage-containing environments should have phage-resistance mutations that are more strongly biased towards knocking out the protein’s native function. Most phages use receptors with non-essential cellular functions, despite essential receptors being slower to evolve phage resistance [2]. Future sequence-function maps should assess the role of missense variation in the acquisition or maintenance of phenotypes in different environments.Mal^−^

## Data Bibliography

1. Raw Illumina sequences have been deposited at the NCBI under the BioProject ID

number PRJNA604031 (2020).

2. Andrews, B., Fields, S., Data from: Distinct Patterns of Mutational Sensitivity for λ

Resistance and Maltodextrin Transport in Escherichia coli LamB. figshare

https://doi.org/10.6084/m9.figshare.c.4837605.v1 (2020).

## Supplementary Data

Supplementary material 1
