# Supplementary material for: Distinct patterns of mutational sensitivity for λ resistance and maltodextrin transport in Escherichia coli LamB
Source: Microb Genom. 2020 Apr 2;6(4):e000364. doi: 10.1099/mgen.0.000364 (PMC7276705; doi:10.1099/mgen.0.000364)
Supplement: Supplementary material 1 [file mgen-6-364-s001.pdf]

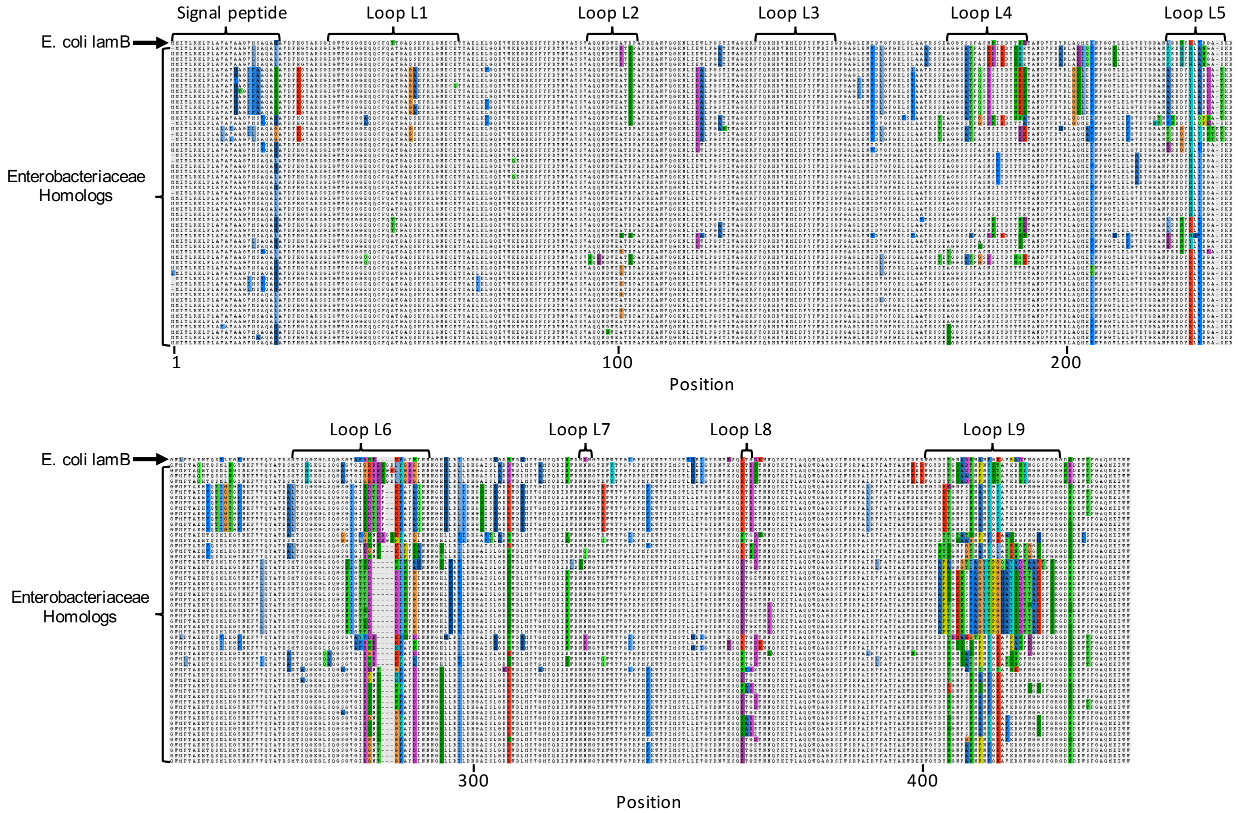

Supplementary Figure 1: Multiple sequence alignment of LamB Homologs. A protein alignment of homologs of LamB from the family Enterobacteriaceae, ranging from 82-97% identical to the query, is shown, with residues that deviate from the consensus residue colored by residue property. The extracellular loops and signal peptide are each annotated, showing a high rate of missense variation in some, but not all, of the extracellular loops, especially Loops L4, L5, L6, and L9. These loops are likely diversified by selection against predation, as they are easily accessible to predators on the extracellular face of the protein.

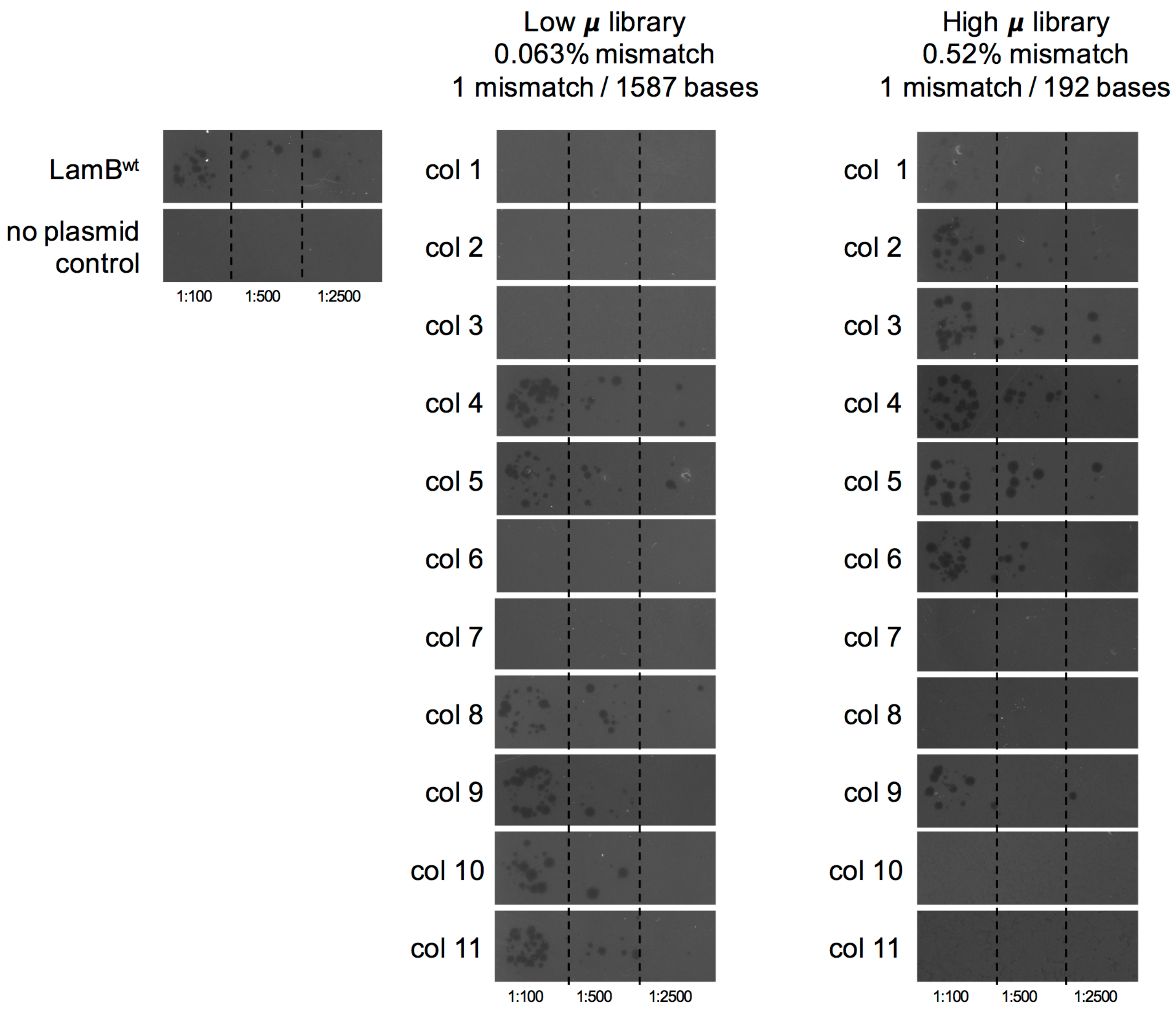

Supplementary Figure 2: Individual phenotyping of randomly chosen variants from the error-prone PCR libraries. Eleven variants from each library were chosen at random by plating on non-selective media and picking colonies for individual assays of λ-sensitivity or maltodextrin transport. Each colony was grown up overnight, back-diluted 1:100 into induction media containing IPTG, then plated in top agar on LB. The indicated dilutions of λ were then spotted onto each plate, using 3 μL of diluted lysate per spot, and the plates were incubated at 37°C overnight before imaging.

Supplementary Figure 3

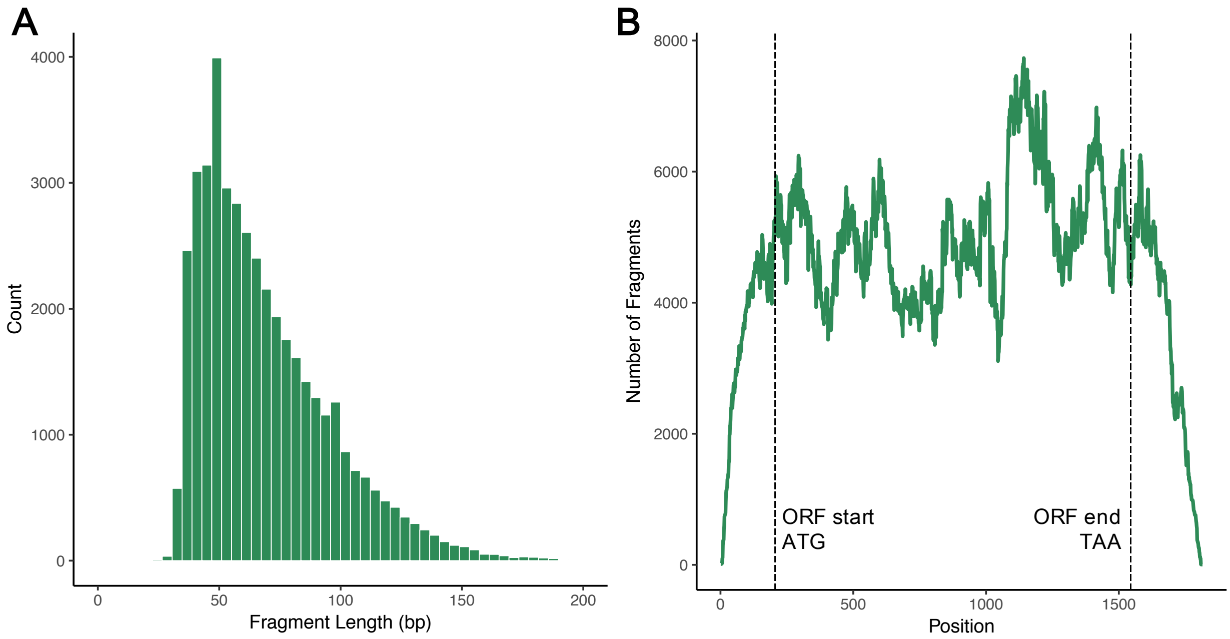

Supplementary Figure 3: Sequencing output from a representative sample. (A) The length distribution of Tn5-generated fragments sequenced from the first replicate of the control population for the λ selection. Lengths shown have had adapter and index sequence trimmed off. (B) Coverage depth, in terms of number of unique fragments, over the length of the amplicon that was sequenced. The *lamB* ORF is indicated, as well as flanking regions that were included due to expected coverage falloffs at the DNA termini.

Supplementary Figure 4

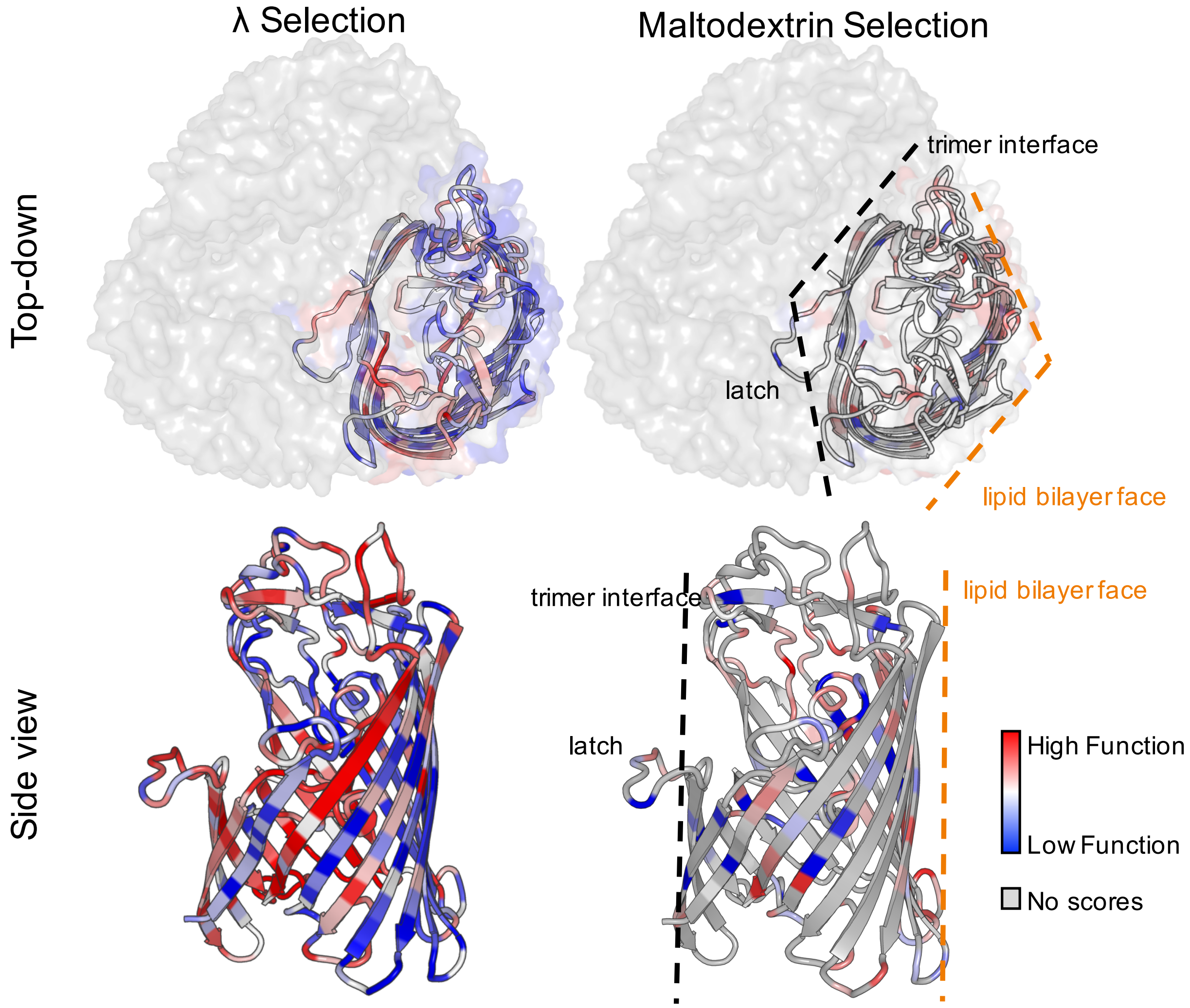

Supplementary Figure 4: Structural components of LamB with respect to assayed phenotypes. The structure of LamB, as determined by x-ray crystallography [12], is shown as viewed from outside the cell (top-down) vs. laterally from within the membrane (side view). In the top-down view, the other monomers of the homotrimer are shown in light grey. In the λ selection, residues that are against the trimer interface generally contain λ-sensitive mutations, while residues facing the lipid bilayer generally contain λ-resistant mutations.

Supplementary Figure 5

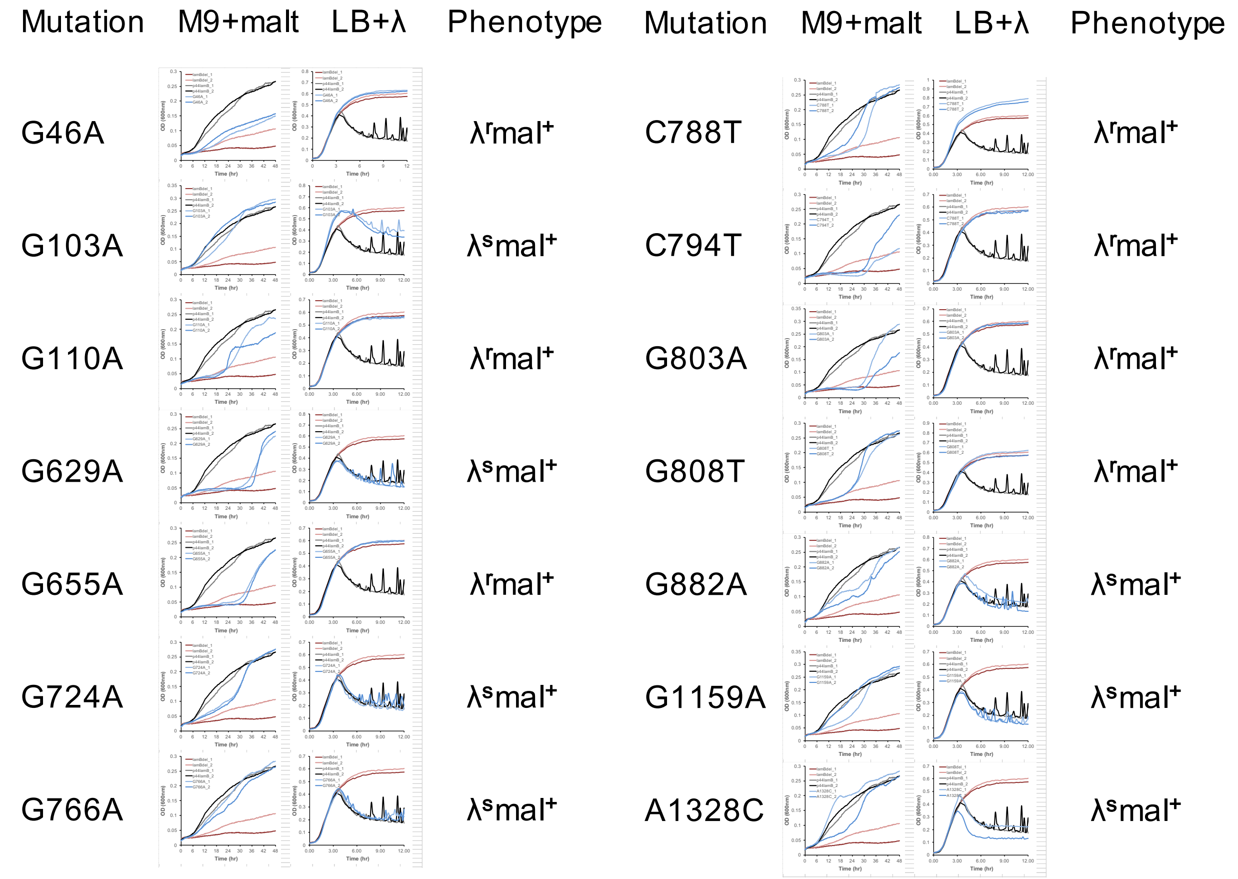

Supplementary Figure 5: Individual growth curve validations of putative $\lambda^r mal^+$ mutations. Mutations cloned into a wild type background were expressed in DH10B(*lamB*$^{\Delta}$) and grown for 48 hours at 37ºC (shaking) in either LB+λ or M9+maltodextrin. During growth, OD_{600} was measured every 10 minutes. OD spikes in cultures that were killed by λ are caused by cellular debris clumping together and do not indicate growth.

Supplementary Figure 6

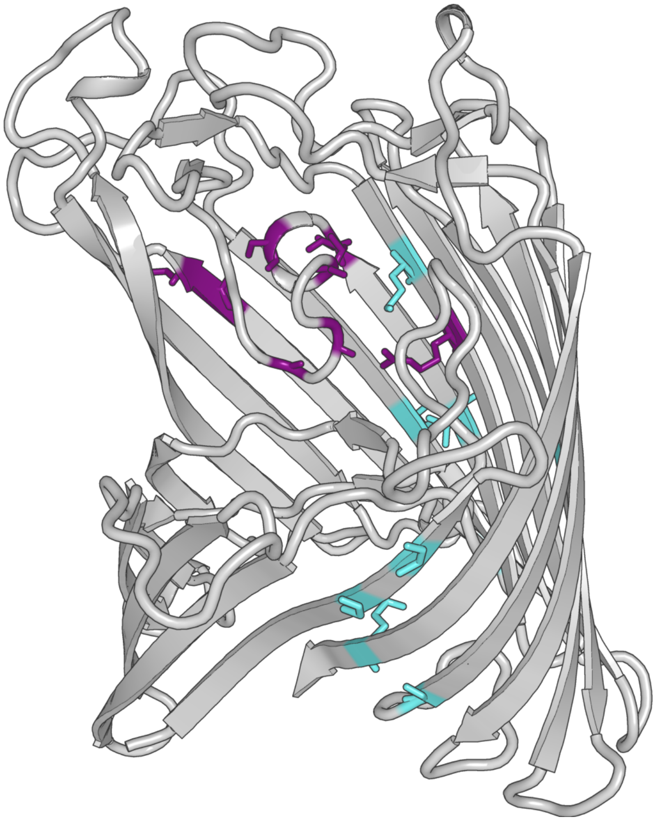

Supplementary Figure 6: Sufficient and Insufficient putative $\lambda^r mal^+$ mutations on the LamB structure. Mutations that were sufficient to confer λ-resistance are shown in purple, and are tightly clustered around Loop L6. Individually assayed mutations that were not sufficient to confer λ-resistance are shown in cyan, and are more dispersed over the protein structure.
